# Kawasaki Shock Syndrome in a 12-Year-Old Girl Mimicking Septic Shock

**DOI:** 10.1155/2016/4949036

**Published:** 2016-12-22

**Authors:** Vindika Prasad Sinhabahu, Janani Suntharesan, Dimuthu Saraji Wijesekara

**Affiliations:** ^1^Professorial Paediatric Unit, Colombo South Teaching Hospital, Kalubowila, Sri Lanka; ^2^Professorial Paediatric Unit, Lady Ridgeway Hospital for Children, Colombo, Sri Lanka

## Abstract

Kawasaki disease is diagnosed when fever lasts for more than 5 days with the presence of four out of five of the following clinical features: bilateral conjunctival congestion, changes in the lips and oral cavity, polymorphous exanthem, changes in peripheral extremities, and acute nonpurulent cervical lymphadenopathy (Nakamura et al., 2012). The average age of onset is 2 years and 90% of patients are below 5 years of age. Boys are more affected than girls (Cox and Sallis, 2009). This case report describes an adolescent female who was initially managed as having septic shock and subsequently found to have Kawasaki shock syndrome.

## 1. Case Report

A 12-year-old girl presented with high spiking fever (103°F) for 3 days with headache, body aches, and an itchy rash for one day. There was no bleeding from gums, skin, urine, or stools. She did not have any urinary or respiratory symptoms. There was no contact history of fever or rash. She had a past history of simple febrile convulsions. She did not give a history of any known allergies, atopy, or asthma.

**Figure 1:**
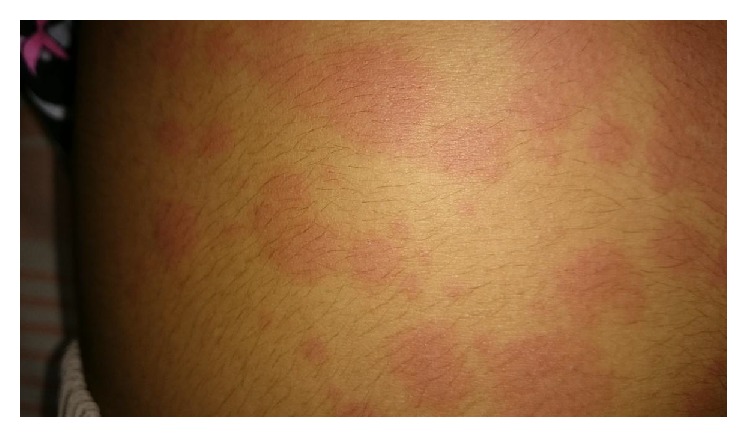
Erythematous itchy rash.

Examination revealed a febrile ill child with cervical lymphadenopathy and urticarial rash over the body which changed in the distribution with fever spikes (Figure 1). Her peripheries were warm and pulse rate was 92/min. Blood pressure was 100/70 mmHg. Respiratory, neurological, and abdominal examinations were normal. There was no clinical evidence of central nervous system infections.

Investigations on admission showed white blood cell (WBC) count of 5580/mm^3^ with 85% neutrophils and platelet (Plt) count of 116000/mm^3^, C-Reactive Protein (CRP) of 161 mg/l, and Erythrocyte Sedimentation Rate (ESR) of 40 mm/1st hour. Summary of investigations is shown in Table 1. Child was initially managed as having viral fever with the possibility of Dengue fever. She was treated with IV cefotaxime as for sepsis when high spiking fever continued with high CRP.

On Day 4 of illness, child developed nonpurulent conjunctivitis with periorbital oedema. She was noted to have haemodynamic instability with hypotension and tachycardia. She received ICU care and needed two inotropes to maintain her haemodynamic stability for 3 days (Day 4–Day 6). As the inotropes were given for short duration, significant side effects were absent. Her urine output was normal throughout the illness. With the presence of fever, rash,and haemodynamic instability, viral myocarditis, toxic shock syndrome, and Steven Johnson syndrome were considered as possible diagnosis compared to atypical Kawasaki disease (KD) with the evolving clinical situation on Day 4 of illness.

2D Echo on Day 8 of illness showed anteroseptal hypokinesia with dilated left anterior descending (LAD) artery with normal Left ventricular and right ventricular function and no pericardial effusion with a negative troponin I and CK-MB. LAD diameter was 2.3 mm with dilated area with a diameter of 3.5 mm. Alanine transaminase (ALT) and aspartate transaminase (AST) were 86 U/l and 115 U/l on Day 4 which persisted above the reference range till Day 16 of illness. Serum bilirubin was 12 mg/dl on Day 4. Ultrasound scan showed bilateral pleural effusions and moderate ascites without hepatosplenomegaly. Repeated blood cultures did not reveal any bacterial growth. Serology for measles, leptospirosis, rubella, Dengue, Epstein-Barr virus, and Polymerase Chain Reaction (PCR) for influenza was negative. ANA was negative. Hypoalbuminemia was noted from Day 4 of illness and persisted for two weeks. CRP was 289 mg/l on Day 5 of illness which came down to 9.2 mg/l on Day 16. Serum Ferritin was 694.5 ng/ml on Day 10 of illness. Intravenous immunoglobulin 2 g/kg over 24 hours was given on Day 8 of illness for the treatment of Kawasaki disease. Aspirin 80 mg/kg in four divided doses was started and continued. Fever settled on Day 10. Ascites and pleural effusions settled subsequently. Two months after the illness, child was asymptomatic and 2D Echo done two months later showed dilated LAD without aneurysm formation.

## 2. Discussion

Initial differential diagnoses in our patient are viral exanthema with myocarditis, systemic lupus erythematosus, toxic shock syndrome, Scarlet fever, systemic onset Juvenile Idiopathic Arthritis (s-JIA), Steven Johnson syndrome, and atypical KD.

KD is an acute febrile illness diagnosed with fever for >5 days with 4 out of 5 of the following signs: bilateral conjunctival congestion, changes in the lips and oral cavity, polymorphous exanthema, changes in peripheral extremities, acute nonpurulent conjunctivitis, and cervical lymphadenopathy [1]. KD is the commonest cause of acquired heart disease in children in USA [2]. Poor prognostic factors include male sex, age < 1 year and >8 years, and fever lasting for >16 days.

Our patient had conjunctivitis, exanthema, and cervical lymphadenopathy which led to the diagnosis of atypical KD. Serious cardiac complications in KD include coronary artery aneurysms, decreased myocardial contractility, congestive heart failure (CHF), arrhythmias, and myocardial ischemia [2]. Heart failure is reported in the febrile phase of KD [1]. The most important laboratory findings in KD were leukocytosis, thrombocytosis, elevated CRP and ESR, hypoalbuminemia, hyperbilirubinaemia, elevated ALT and AST, and sterile pyuria [3, 4]. Urticarial exanthema and CRP > 10 mg/dl were identified for risk factors for coronary aneurysms which were present in our patient [4].

Toxic shock syndrome will have similar presentation with hypotension and elevated ALT and conjunctivitis but coronary artery dilation is unlikely in it [5]. Pleural effusions can occur due to concurrent infections like* Mycoplasma *and* Streptococcus* [6, 7]. Increased microvascular permeability is seen in vasculitis of KD which may account for periorbital oedema [8].

Kawasaki shock syndrome (KSS) characterized by systolic hypotension or clinical signs of poor perfusion is found in 6% of KD patients [9]. It was a retrospective diagnosis in our patient.

One study showed serum ferritin levels were significantly elevated in s-JIA patients compared with KD patients with a cut-off value of 369.6 ng/ml [10]. It was not helpful in differentiation in our patient.

## Figures and Tables

**Table 1:** Summary of investigations.

Day of illness	Day 3	Day 4	Day 5	Day 6	Day 7	Day 8	Day 10	Day 13	Day 20
WBC (mm^3^)	9640	5580	6120	4500	12790	13830	20150	18800	18910
N (%)	78	85	83	89.7	91	85	85.2	79.8	56.5
L (%)	20	11	8.6	7.3	5.6	8.6	10	11	36.5
Plt (10^3^/mm^3^)	178	116	110	105	133	130	245	385	709
AST (U/l)		78.5		115	123	81	64	128	
ALT (U/l)		62.1		86.3	85.4	71	48	68.7	
CRP (mg/l)	107	161	289	251			122		9.3
Albumin (g/l)					23.8	23.5	23.4		
Sodium (mmol/l)					139	138	138		

## References

[1] Nakamura Y., Yashiro M., Uehara R. (2012). Epidemiologic features of Kawasaki disease in Japan: results of the 2009-2010 nationwide survey. *Journal of Epidemiology*.

[2] Cox J. R., Sallis R. E. (2009). Recognition of Kawasaki disease. *The Permanente Journal*.

[3] Sato Y. Z., Molkara D. P., Daniels L. B. (2013). Cardiovascular biomarkers in acute Kawasaki disease. *International Journal of Cardiology*.

[4] Caballero-Mora F. J., Alonso-Martín B., Tamariz-Martel-Moreno A., Cano-Fernández J., Sánchez-Bayle M. (2011). Kawasaki disease in 76 patients. Risk factors for coronary artery aneurysms. *Anales de Pediatría*.

[5] Tanner M. H., Pierce B. J., Hale D. C. (1981). Toxic shock syndrome. *Western Journal of Medicine*.

[6] Lee M. N., Cha J. H., Ahn H. M. (2011). Mycoplasma pneumoniae infection in patients with Kawasaki disease. *Korean Journal of Pediatrics*.

[7] Alhammadi A., Hendaus M. (2013). Comorbidity of Kawasaki disease and group A streptococcal pleural effusion in a healthy child: a case report. *International Journal of General Medicine*.

[8] Terai M., Honda T., Yasukawa K., Higashi K., Hamada H., Kohno Y. (2003). Prognostic impact of vascular leakage in acute Kawasaki disease. *Circulation*.

[9] Taddio A., Rossi E. D., Monasta L. (2016). Describing Kawasaki shock syndrome: results from a retrospective study and literature review. *Clinical Rheumatology*.

[10] Mizuta M., Shimizu M., Inoue N. (2016). Serum ferritin levels as a useful diagnostic marker for the distinction of systemic juvenile idiopathic arthritis and Kawasaki disease. *Modern Rheumatology*.

